# High Density Infill in Cracks and Protrusions from the Articular Calcified Cartilage in Osteoarthritis in Standardbred Horse Carpal Bones

**DOI:** 10.3390/ijms16059600

**Published:** 2015-04-28

**Authors:** Sheila Laverty, Mathieu Lacourt, Chan Gao, Janet E. Henderson, Alan Boyde

**Affiliations:** 1 Comparative Orthopaedic Research Laboratory, Département de Sciences Cliniques, Faculté de Médecine Vétérinaire, Université de Montréal, C.P. 5000, Saint-Hyacinthe, QC J2S-7C6, Canada; E-Mails: sheila.laverty@umontreal.ca (S.L.); matlacourt@gmail.com (M.L.); 2 Division of Experimental Medicine, McGill University, Montreal, QC H3G-1A4, Canada; E-Mail: chan.gao@mail.mcgill.ca; 3 Bone Engineering Labs, Research Institute-McGill University Health Centre, Montreal General Hospital C9-133, 1650 Cedar Ave, Montreal, QC H3G-1A4, Canada; E-Mail: Janet.henderson@mcgill.ca; 4 Dental Physical Sciences, Oral Growth and Development, Dental Institute, Barts’ and The London School of Medicine and Dentistry, Queen Mary University of London (QMUL), London E1 4NS, UK

**Keywords:** cartilage, bone, osteoarthritis, X-ray microtomography, dystrophic calcification, chondrocyte apoptosis, zone of calcified cartilage

## Abstract

We studied changes in articular calcified cartilage (ACC) and subchondral bone (SCB) in the third carpal bones (C3) of Standardbred racehorses with naturally-occurring repetitive loading-induced osteoarthritis (OA). Two osteochondral cores were harvested from dorsal sites from each of 15 post-mortem C3 and classified as control or as showing early or advanced OA changes from visual inspection. We re-examined X-ray micro-computed tomography (µCT) image sets for the presence of high-density mineral infill (HDMI) in ACC cracks and possible high-density mineralized protrusions (HDMP) from the ACC mineralizing (tidemark) front (MF) into hyaline articular cartilage (HAC). We hypothesized and we show that 20-µm µCT resolution in 10-mm diameter samples is sufficient to detect HDMI and HDMP: these are lost upon tissue decalcification for routine paraffin wax histology owing to their predominant mineral content. The findings show that µCT is sufficient to discover HDMI and HDMP, which were seen in 2/10 controls, 6/9 early OA and 8/10 advanced OA cases. This is the first report of HDMI and HDMP in the equine carpus and in the Standardbred breed and the first to rely solely on µCT. HDMP are a candidate cause for mechanical tissue destruction in OA.

## 1. Introduction

Articular cartilage comprises two layers mainly distinguished by calcification of the one to make the other: the deep layer of hyaline articular cartilage (HAC) becomes articular calcified cartilage (ACC) [1,2]. This mineralization both stiffens the material and allows it to be resorbed by osteo(chondro)clasts to permit scaffolded bone formation upon the eroded surface, which attaches bone tissue to cartilage, absolutely central to the function of bones and joints. The ultrastructural grain of deep HAC and all ACC is dominated by the surface-normal orientation of the cartilage collagen. The underlying subchondral bone (SCB) collagen, on the contrary, is deposited as a layered mat with fibers parallel to the resorbed interface and, thus, better organized to prevent cleaving and cracking. The highly anisotropic and stiff ACC is thus predestined to cleave perpendicular to the joint surface, there being nearly no elements to resist this [3]. Cracks in the ACC layer may extend into SCB [3,4]. In both layers, they may be removed by osteoclastic removal in the periodic repair, replacement and refreshment of this junction [2,5]. However, such cracks also repair spontaneously by the formation of a high-density mineralized infill (HDMI) phase. This was first found using backscattered electron scanning electron microscopy (BSE SEM) of flat surfaces of polymethylmethacrylate (PMMA)-embedded blocks in studies of Thoroughbred racehorse distal third metacarpal bones (Mc3: fetlock [6,7]) and hock joints [6]. It should be noted that BSE SEM has much higher spatial resolution than microradiography (X-ray microscopy) or micro-computed tomography (µCT). It is not yet known what the nature of the organic matrix in HDMI is, but it is clear that there is very little of it, since the mineral content is so high, so that the matrix must be “watery”. BSE SEM images of corresponding macerated preparations retaining the 3D morphology of the mineralizing front (MF) surface of the ACC showed that the HDMI phase extended from cracks and that parts of it projected a little into the HAC space, above the level of the MF of the ACC [6].

Ferguson *et al.* [8] studied human OA femoral heads with quantitative BSE SEM correlated with nano-indentation measurement. They showed high-density fragments impacted into the eburnated surfaces. The stiffness modulus of these features was substantially increased above values found in reference ACC and SCB. These authors speculated that such high-density fragments might be involved as a kind of cutting and grinding compound in HAC destruction in OA.

Prominent protrusions of high-density material (HDMP) were found in the palmar/plantar regions of distal metacarpal/metatarsal condyles in association with palmar/plantar osteochondral disease (POD) in racing Thoroughbred racehorses [9]. This study used correlated confocal autofluorescence LM to show their location within HAC and BSE SEM imaging to show their origin at the ACC MF and continuity with HDMI in cracks in ACC, which can extend as splits or tears in HAC and come to contain a similar high-density infill. The tendency of these HDMP to fragment was amply illustrated. It was also shown that there is little residual matrix left after decalcification and that there was no cellular content within the protrusions. In this respect, there is a strong similarity between HDMP in HAC and a subset of densely-mineralized features in dystrophic soft tissue calcification, namely in those regions in which pre-existing cells and extracellular matrix components are not used as a matrix [10].

HDMP occupying practically the full thickness of HAC have recently been described in the tarsal joints of Icelandic horses, a breed that constitutes a good genetic “model” of OA [11]. In this instance, some of the HDMP had been detected at clinical imaging-scale resolution using both X-ray computed tomography (CT) and magnetic resonance imaging (MRI) before the bones were sectioned. Further detailed study used correlated digital microradiography, µCT, autofluorescence confocal LM and BSE SEM before and after iodine staining, the latter allowing the study of soft tissue histology directly in the SEM.

Human HDMP were discovered by a full high spatial and high contrast resolution µCT scan of an entire OA femoral head [12]. Retrospectively, it was found that the same features could be seen in 3 Tesla MRI dual echo steady state sequence (DESS) scans which had been recorded before the µCT. Sectioning the samples for preparation for digital microradiography, higher resolution µCT and BSE SEM before and after iodine staining was guided by information from the initial µCT scan. A few other cases studied only by MRI showed similar features. In this human study [12] and in all previous studies of HDMI and HDMP in joints, PMMA embedding and tedious sample preparation had been used to prove their existence, whereas it might have been sufficient to use the lower resolution, but 3D imaging, provided by µCT.

HDMP thus represent the *de novo* formation of a dead tissue within living cartilage by an acellular mechanism and, as such, merit special consideration in a journal issue devoted to the general topic of death in cartilage. 

Lacourt *et al.* [4] used visual pathology grade scoring, histopathology and µCT to study OA in the carpal bones of Standardbred racehorses, where the etiology involves repetitive overload exercise similar to POD in Thoroughbreds. Standard numerical analyses for the 3D µCT data and a description of morphological findings, such as obvious pits, made from the initial 3D renderings were given in the 2012 paper [4]. In the present study, we re-examined this existing µCT data to see whether localized high density features could be discovered. At the time of the initial study, the authors were interested in the striking subchondral bone porosity visible on µCT and cracks in the calcified cartilage in co-localized Safranin O Fast green-stained paraffin sections of demineralized blocks. They had not focused on the possibility of spotting accumulations of small, but unusually radio-dense patches within the ACC on the µCT images. Indeed, at a nominal voxel resolution of 20 µm, which is 8000 µm^3^, it might not be obvious that features as small as “cracks” could be spotted.

## 2. Results

HDMI and HDMP were found with the greatest incidence in the samples classified as advanced OA, less in the early OA and only one HDMP in a control sample.

### 2.1. Spotting Dense Regions in Noisy µCT Image Stacks

Rapid scanning up and down in Z through the entire *XY* data stacks using, for example, the browse feature in Jasc Software Products Paint Shop Pro5 with the largest (120 pixel wide) “thumbnails” brought likely high-density regions to attention very rapidly. Loading the entire datasets as virtual stacks in ImageJ equally provided a rapid means of spotting likely areas.

### 2.2. Examples of Single Images Demonstrating High-Density Infill in ACC: HDMI

**Figure 1:**
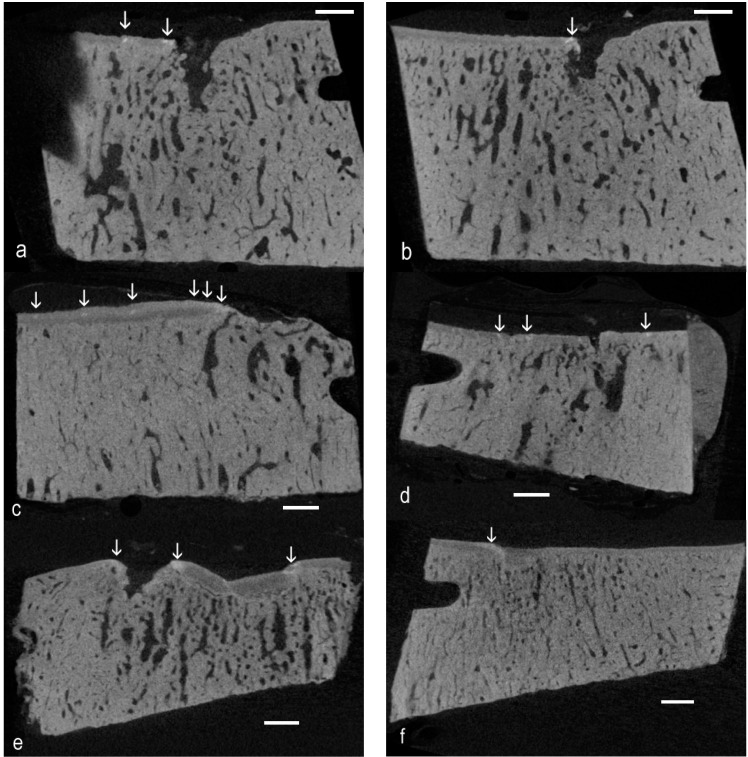
Unprocessed single *XY* plane images from Z stacks in which there are localized patches (vertical arrows) with higher density below the level of the mineralizing front of the articular calcified cartilage (ACC). Scale bars = 1 mm. (**a**) Eight-year-old, which showed the highest number of easily-spotted high-density mineral infill (HDMI) fields in a control sample; (**b**) Parallel slice at 360 µm; (**c**) Advanced OA, five-year-old; (**d**) Advanced OA, four-year-old; (**e**) Early OA, two-year-old; and (**f**) Early OA, same two-year-old, but in the more palmar core.

Figure 1 shows unprocessed single *XY* plane images from Z stacks in which there are localized patches with higher density below the level of the mineralizing front (MF) of the ACC. Examples are also shown where there is a local elevation of the MF with no noticeable change in the grey level, so that although it is a protrusion, it is not obviously hyperdense.

### 2.3. Single Images Demonstrating High Density Infill Proud of the ACC MF: HDMP

**Figure 2:**
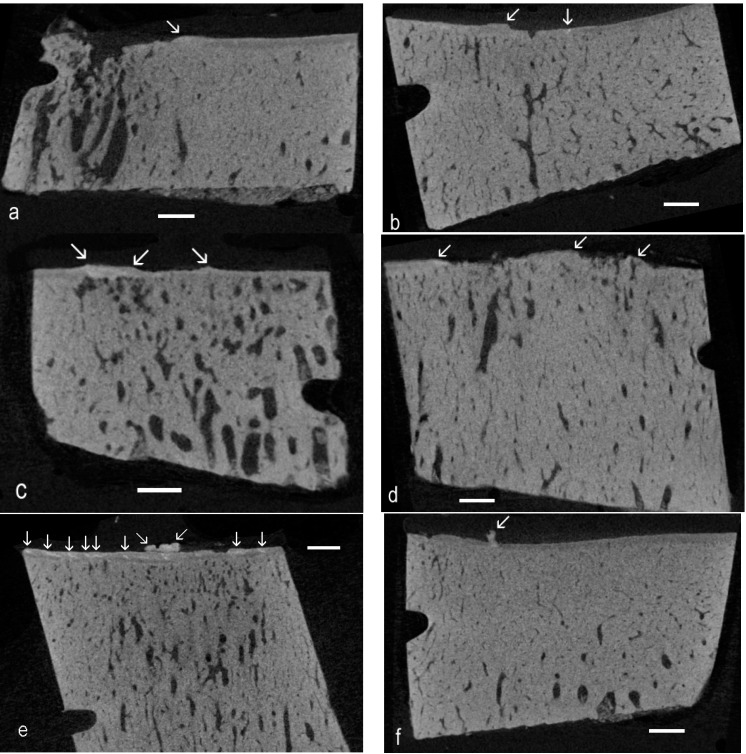
Unprocessed single *XY* plane images from Z stacks with localized patches (oblique arrows) with higher density proud of the level of the mineralizing front (MF) of the ACC. (**a**) Advanced OA, seven-year-old; (**b**) Advanced OA, four-year-old; (**c**) Early OA, two-year-old; (**d**) Advanced OA, seven-year-old; (**e**) Early OA, six-year-old; and (**f**) Advanced OA, seven-year-old.

High-density mineralized protrusions are those which project the proud of the MF of the ACC, and examples are shown in Figure 2.

### 2.4. Improving Confidence in Identifying Hyperdense Regions in Noisy µCT Image Stacks

We define “hyperdense” on the basis that image grey levels are “whiter” (the mineral content is higher) than is found in surrounding ACC, in which values are usually above those in SCB. Whereas protrusions (oblique arrows in Figure 2) may be spotted on the basis of morphology, high-density inclusions (vertical arrows in Figure 1 and Figure 2) in ACC are more difficult to see in single image planes. However, *XY* plane images adjacent in Z can be averaged to provide mean values, greatly increasing confidence in spotting above normal values.

**Figure 3:**
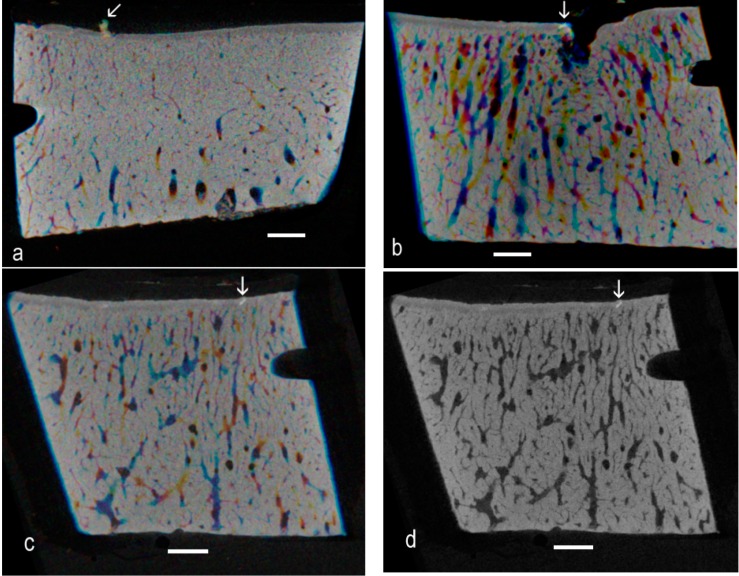
(**a**) Successive eight-bit grey level images (averages of two original Z levels) images assigned as Red, Green and Blue: matching brighter patches show as whiter against a colored background. Advanced OA, seven-year-old; (**b**) From sample from an eight-year-old that showed the highest number of easily-spotted HDMI fields in a control sample. Successive averages of four grey level images displayed as R, G and B components in a composite image; (**c**) Another region from the same sample; six sequential Z planes summed in pairs, then pairs displayed as RGB components; and (**d**) Same six planes processed to retain the darkest pixels.

Successive images can be displayed as red, green and blue components in a composite image, when matching brighter patches show as white against a colored background. Likewise, images can be averaged over depths of, say, 2, 3 or 4 original Z levels, and these averaged images are assigned as R, G and B components in a composite image (Figure 3a–c). Another simple strategy for spotting high-density patches is to use image arithmetic to look for the darkest regions in a grey level Z series (Figure 3d).

### 2.5. Volume Rendering

**Figure 4:**
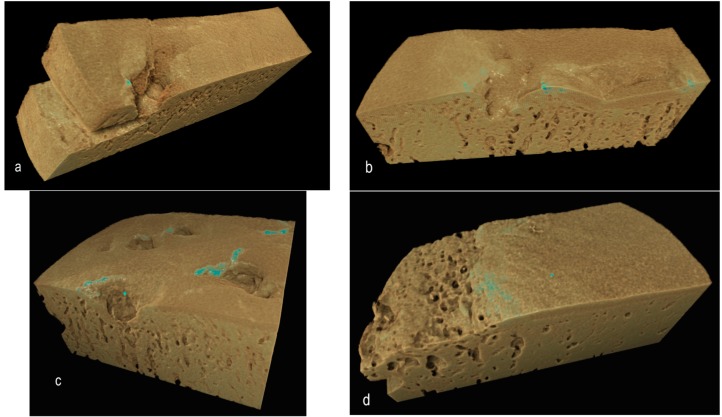
Drishti renderings showing high-density regions within or at the ACC MF surface, the denser regions tinged blue: (**a**) Two-year-old, left side, early OA in more palmar site; (**b**) Same bone, the more dorsal site, early OA; (**c**) Same horse, right limb, more dorsal site, advanced OA; and (**d**) Four-year-old, more dorsal site, advanced OA.

**Figure 5:**
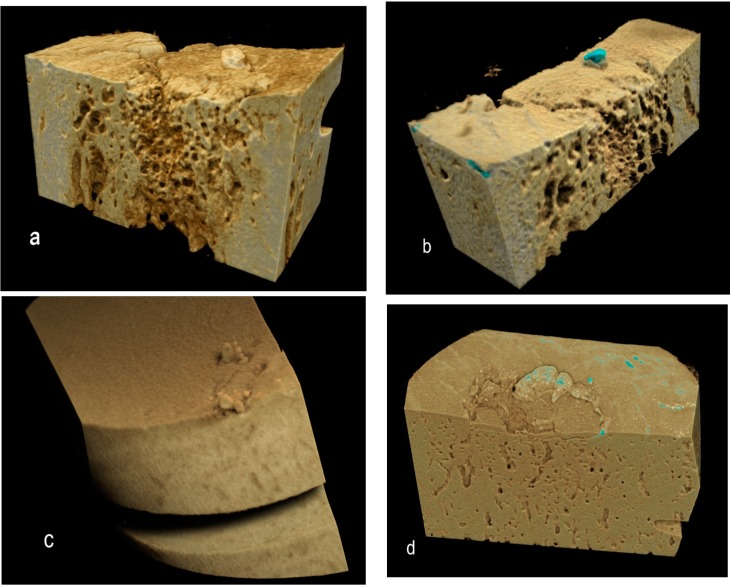
Drishti renderings showing high-density mineralized protrusions from the ACC MF: (**a**,**b**) Four-year-old, advanced OA; (**c**) Advanced OA, seven-year-old; and (**d**) Early OA, six-year-old.

Rendering the 3D volumes (in our case, using Drishti) is another good method for averaging data to show the distribution of microscopic domains defined by thresholding and for showing the morphology of frank lesions (Figure 4), demonstrating the extent and the prominence of protrusions (Figure 5).

## 3. Discussion

The work of Radin and Rose [2] has received a great deal of attention in the OA field. They proposed that an increase in the stiffness gradient in SCB may both initiate and favor the progression of HAC damage in OA. They held that stresses at the base of the articular cartilage could cause deep horizontal splits in that tissue, whilst, in fact, observation shows that such splits are mainly vertical. Burr and Radin [5] further developed the idea that microcracks in the subchondral plate might contribute to degeneration of the hyaline cartilage by causing vascular invasion of ACC and an advance in the tidemark mineralising front of the ACC: the consequent thinning of the HAC was purported to increase shear stresses beyond the point at which the cartilage could still repair itself, and hence, result in cartilage degeneration.

Mori *et al.* [13] described vertical cracks in human femoral head ACC in 150-µm ground sections stained with basic fuchsine. Villanueva *et al.* [14] studied both 100-µm ground sections of human femoral heads prestained with the Villanueva mineralized bone stain, undecalcified, plastic-embedded, 5–15 µm microtome sections stained with the Villanueva gallocyanin chrome alum blood stain methods and, also, found microcracks in the subchondral bone and at the osteochondral junction. Neither of these studies could address the issue of HDMI. Studies based on demineralised sections [1,15] correctly spotted vertical cracks in ACC, but could not determine whether there might have been a mineralised material within them. Indeed, none of these studies considered that possibility.

Norrdin and Stover [16] studied POD in the distal third metacarpal bones of Thoroughbred racehorses using plain radiography and secondary electron (SE) SEM imaging of polished 4-mm slice surfaces. They showed gross cracking starting in the volumetrically-densified subchondral bone formed in response to overload exercise. Their samples were treated with bleach, which leaves a brittle surface, so that polishing will damage crack edges, and the use of gold coating and SE imaging will have prevented them from seeing the hyperdense phase, which must have been present. It is important to use embedded material and the compositional (atomic number) contrast from the BSE SEM mode to study HDMI.

The present re-examination of archival µCT data [4] proved the existence of high-density material within the ACC domain in OA in the Standardbred racehorse carpus. There is sufficient evidence from the 3D rendered data to show the linear or elongated nature of such patches, allowing us to conclude that they are cracks repaired by the intercalation of densely-mineralised matrix as exemplified in BSE SEM studies [6,7,9]. The interest here is that the findings extend the range of equine joints and equine breeds in which this phenomenon has been shown to occur, showing many examples and that the demonstration was made purely using µCT, at the rather poor “micro” resolution of only 20 µm. This gives rise to the hope that other investigators may have existing µCT datasets that can be more carefully scrutinised to extend the discoveries of HDMI into other joints and other species.

Further, this study has revealed many instances of HDMP. Where these have been studied at high resolution by BSE SEM, it is evident that these features may fragment *in situ* to produce sharp-edged fragments, which could wreak havoc within the HAC [9,11,12]. Obviously, the greatly improved resolution of BSE SEM at about 0.1 µm provides more details of the histopathology, and this can be combined with iodine staining to show the soft tissue histology in context [11,12]. Given the high frequency of HDMI and HDMP observed in both early osteoarthritis (EOA) and advanced osteoarthritis (AOA) in the present study (Table 1), it would be worthwhile to obtain more material for further study. By macerating the isolated bones to remove HAC and leave tissue below the ACC MF, but retaining any HDMP, we would be able to map these features across entire joint surfaces using controlled vapor pressure mode SEM [11]. Higher spatial resolution µCT would also be desirable to map and discriminate both HDMI and HDMP, but, as in the foregoing, a main purpose here was to check on the value of the nominal 20-µm voxel data. It would also be invaluable to have available high contrast resolution µCT as used in the only human study to date [12].

**Table 1:** Number of images of clusters with HDMI or HDMP spotted by code blind observer. D = Droite = Right. G = Gauche = Left: Last digit 1 = more dorsal 2 = more palmar site. Assignment to Control, EOA and AOA groups was done at the time of the post mortem acquisition of the samples.

ID #	Age	Number	ID #	Age	Number	ID #	Age	Number
Controls			Early OA			Advance OA		
C1G1	8	13	C4G1	7	0	C5D1	2	4
C1G2	8	0	C4G2	7	0	C5D2	2	0
C2G1	4	0	C5G1	2	6	C21D1	7	4
C2G2	4	0	C5G2	2	5	C21D2	7	1
C3D1	7	2	C10D2	7	4	C21G1	7	4
C3D2	7	0	C19G1	6	7	C21G2	7	0
C13G1	8	0	C19G2	6	3	C40D1	5	17
C13G2	8	0	C38D1	5	12	C40D2	5	7
C20G1	6	0	C38D2	5	0	C44D1	4	8
C20G2	6	0				C44D2	4	5

HDMP have been seen in high-resolution post-mortem CT and MRI imaging of equine tarsal [11] and MRI imaging of human hip joints [12] and could probably be imaged *in vivo*. However, do the HDMP we consider have any correspondence with features known from *in vivo* clinical imaging in OA? The most probable correlate is with the so-called central osteophytes (CO) [11,17,18,19,20]. Varich *et al.* [17] found central excrescences to occur in 62 of 66 cases classified as OA out of a total of 100 anatomical specimens. McCauley *et al.* [18] found a 15% prevalence of central osteophytes in human knee on MR imaging: see also the good images in Gold *et al.* [19]. COs were present in 35% of equine metacarpal bones [20]. As for whether COs = HDMPs or whether COs seen by clinical MRI are just the larger end of the size range, we know of only one study to date that looked for a correlation with histology [20]. In that study, COs showed as focal hypointense protuberances from the subchondral plate into the cartilage with 1.5 Tesla MRI spoiled gradient recalled (SPGR) sequences. Histologically, they were composed of bone reaching the proud of ACC MF, the bone projection being capped by a new calcified cartilage domain [20]. The HDMP we show in the present study are therefore probably not the same thing.

## 4. Materials and Methods

**Figure 6:**
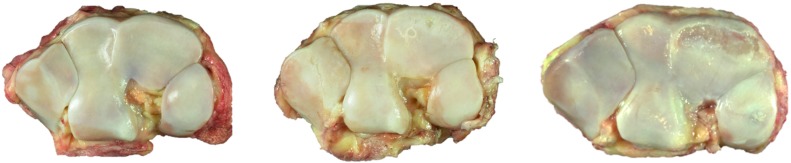
Third carpal bones, dorsal to top: **Left**, control; **Center**, early OA; and **right**, advanced OA.

Tissue was harvested at a local abattoir from Standardbred racehorses with a previous racing career, as previously described [4]. A scoring system of the macroscopic cartilage degeneration in the third carpal bones was used to create control (C), early osteoarthritis (EOA) and advanced OA (AOA) groups. Briefly, control (C) specimens had no visible cartilage macroscopic lesions; EOA specimens had fissures and or partial thickness in an area less than one square centimeter or advanced osteoarthritis (AOA) with partial to full thickness erosions/ulcerations in an area greater than one square centimeter (Figure 6).

**Figure 7:**
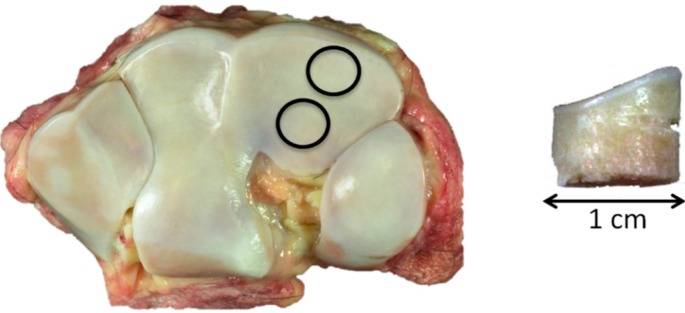
**Left**, control, showing the 1-cm core sample location, dorsal top; and **right**, core sample with dorsal notch.

Two 1-cm diameter osteochondral cores were harvested, one from the more dorsal common location for focal OA lesions in this bone and the second from a more palmar site that is rarely affected (Figure 7 left). The dorsal aspect of each core was notched for orientation (Figure 7 right).

Samples were fixed in 4% fresh formaldehyde (from paraformaldehyde) for 24 h and stored in PBS until µCT imaging using a Skyscan 1172 system (Skyscan (now Bruker), Antwerp, Belgium). Scans were performed using 70 kV and 140 µA and a 0.5-mm Al filter, with a nominal 20-µm isotropic voxel resolution.

In the re-examination of the data presented here, single images were studied using Paint Shop Pro 5 (Jasc Software Products, Eden Prairie, MN, USA) and ImageJ (National Institutes of Health, Bethesda, MD, USA). Volumetric rendering used Drishti software (Australian National University, Canberra, Australia).

## 5. Conclusions

We have found both HDMI and HDMP in equine third carpal bones, studying the Standardbred racehorse breed. This is the first study discovering these features to rely solely on µCT methodology. It should be borne in mind that larger HDMP may be seen in high-resolution MRI imaging [12]. These features should be sought in future studies of the mechanisms of cartilage degeneration and destruction in OA. They may possibly be widespread. They escape attention from the standard methods of histopathology, because the mineral content disappears during decalcification. They are too small to show on routine radiography. They are also too small to be found with standard commercial µCT systems used with whole bones in the size range studied here.
